# Optimal Selection of Imaging Examination for Lymph Node Detection of Breast Cancer With Different Molecular Subtypes

**DOI:** 10.3389/fonc.2022.762906

**Published:** 2022-07-13

**Authors:** Mingjie Zheng, Yue Huang, Jinghui Peng, Yiqin Xia, Yangyang Cui, Xu Han, Shui Wang, Hui Xie

**Affiliations:** Department of Breast Surgery, The First Affiliated Hospital With Nanjing Medical University, Nanjing, China

**Keywords:** breast cancer, molecular subtype, lymph node assessment, imaging examination, ROC (receiver operating characteristic) analysis

## Abstract

**Objective:**

Axillary lymph node management is an important part of breast cancer surgery and the accuracy of preoperative imaging evaluation can provide adequate information to guide operation. Different molecular subtypes of breast cancer have distinct imaging characteristics. This article was aimed to evaluate the predictive ability of imaging methods in accessing the status of axillary lymph node in different molecular subtypes.

**Methods:**

A total of 2,340 patients diagnosed with primary invasive breast cancer after breast surgery from 2013 to 2018 in Jiangsu Breast Disease Center, the First Affiliated Hospital with Nanjing Medical University were included in the study. We collected lymph node assessment results from mammography, ultrasounds, and MRIs, performed receiver operating characteristic (ROC) analysis, and calculated the sensitivity and specificity of each test. The C-statistic among different imaging models were compared in different molecular subtypes to access the predictive abilities of these imaging models in evaluating the lymph node metastasis.

**Results:**

In Her-2 + patients, the C-statistic of ultrasound was better than that of MRI (0.6883 vs. 0.5935, p=0.0003). The combination of ultrasound and MRI did not raise the predictability compared to ultrasound alone (p=0.492). In ER/PR+HER2- patients, the C-statistic of ultrasound was similar with that of MRI (0.7489 vs. 0.7650, p=0.5619). Ultrasound+MRI raised the prediction accuracy compared to ultrasound alone (p=0.0001). In ER/PR-HER2- patients, the C-statistics of ultrasound was similar with MRI (0.7432 vs. 0.7194, p=0.5579). Combining ultrasound and MRI showed no improvement in the prediction accuracy compared to ultrasound alone (p=0.0532).

**Conclusion:**

From a clinical perspective, for Her-2+ patients, ultrasound was the most recommended examination to assess the status of axillary lymph node metastasis. For ER/PR+HER2- patients, we suggested that the lymph node should be evaluated by ultrasound plus MRI. For ER/PR-Her2- patients, ultrasound or MRI were both optional examinations in lymph node assessment. Furthermore, more new technologies should be explored, especially for Her2+ patients, to further raise the prediction accuracy of lymph node assessment.

## Introduction

The progression of breast cancer is characterized by metastasis (1). The presence of regional lymph node metastasis in cancer patients correlates with dissemination to distant organs and a poorer prognosis (1). For breast cancer, modern strategies of axillary lymph node management involve stepwise approaches including fine needle aspiration or core needle biopsy, sentinel lymph node biopsy (SLNB), and axillary lymph node dissection (ALND). Based on preoperative imaging evaluation of axillary lymph nodes, clinicians take corresponding measures. Historically, ALND was regarded as the most accurate method for assessing regional metastatic spread (2). However, associated complications such as seroma, nerve injury, and lymphedema would bring unnecessary pain for pathologically node-negative patients (2, 3). Conversely, residual axillary disease would bring regional recurrence and a poorer prognosis. Therefore, accurate preoperative imaging evaluation of axillary lymph node status is of great importance for precision treatment of breast cancer patients.

Breast cancer is a highly heterogeneous disease. Based on gene expression profiles, it is currently categorized into three distinct molecular subtypes, including HER2 positive (Her2+), ER/PR positive/HER2 negative (ER/PR+Her2-), and triple-negative (ER/PR-Her2-) types (4). Molecular subtype classification of breast cancer is a regular process for individualized cancer management. Distinct molecular subtypes confer different treatment programs and different clinical prognosis (5). Moreover, some reports have indicated that characteristic imaging manifestation was also correlated with the three subtypes mentioned above. For instance, Wang et al. found that compared to HER2-positive breast cancer, HER2-negative breast cancer was more likely to have spiculated margins (6). However, the influence of breast cancer subtypes on the diagnostic performance of axillary imaging is unknown. This raised the speculation that the accuracy of imaging assessment of axillary lymph node metastasis might also be affected by the molecular subtype of primary tumors.

Therefore, in order to determine whether the imaging diagnostic performance of lymph nodes differ among various subtypes of breast cancer, we conducted a retrospective matched cohort study in 2,340 patients, with the goal to provide a more reliable imaging evaluation of lymph node status for each breast cancer subtype.

## Material and Methods

### Patient Population and Data Collection

Patients diagnosed with primary invasive breast cancer and positive axillary lymph nodes after breast surgery between 2013 and 2018 in Jiangsu Breast Disease Center, the First Affiliated Hospital with Nanjing Medical University were included in the study. Exclusion criteria were as follows: male breast cancer, patients without any imaging lymph node staging before surgery [i.e., mammography, ultrasound, breast magnetic resonance imaging (MRI)], and patients whose receptor status was missing. Then, the controls were age- and molecular subtype-matched to the cases, whose axillary lymph node were confirmed negative by surgery. The selection procedure is summarized in **Figure S1**. Data on patients, tumor characteristics, imaging, and histopathological outcome of the axillary lymph nodes were retrospectively collected. The study was approved by the Ethics Committee of Nanjing Medical University.

### Clinical Nodal Status

Pre-operative nodal status was assessed by mammography, ultrasound, and MRI. The imaging results we adopted were performed in all of our patients before local or systemic treatment, including mass puncture biopsy. Mammography were obtained by clinical full-field digital mammography unit, which used molybdenum for target and filter (Selenia, Hologic, USA) (7). Lymph nodes considered abnormal had a size>2cm, increased density, rounded or irregular shape, spiculate margins or the absence of fatty hilum (8) (**Figure S2**). Ultrasound was performed using MyLab Twice (Esaote S.p.A., Genova, Italy) Color Doppler with a 4-13MHz linear transducer (iU22; Philips Medical Systems, Bothell, WA, USA) (8). A lymph node was considered abnormal if the cortex was either focally or diffusely thickened (> 3 mm thick) and the fatty hilum was deformed or absent (**Figure S3**). MRI was conducted using a bilateral eight-channel phased-array breast coil with a 3.0 T scanner (MAGNETOM Trio, Siemens, Germany) to obtain images (9). A positive lymph node was defined as: an irregular contour compared with the contralateral axilla, a node measuring greater than 1 cm, the thickened cortex was >3 mm or there was a loss of fatty hilum (10) (**Figure S4**).

The axillary images *via* mammography, ultrasound, or MRI was interpreted independently by one of five dedicated breast radiologists with more than 5 years of experience in breast imaging.

### Axillary Lymph Node Management

Patients clinically diagnosed with negative nodes underwent SLNB. The SLNB procedure was performed using both the gamma probe to detect radioactivity and blue dye to detect lymphatic vessels. If one or more sentinel lymph nodes were confirmed with macro-metastasis, a completion ALND was then performed. In clinically node positive patients an ALND was performed directly.

### Pathological Assessment of Axillary Lymph Node

SLNB samples were assessed by immediate frozen section and hematoxylin and eosin (H&E) staining. Then the lymph node was subsequently submitted for permanent section and stained with cytokeratin immunohistochemical (IHC), while all ALND samples were embedded in paraffin as permanent section for histological evaluation. Lymph nodes with isolated tumor cells were also considered node-negative and no additional lymph node surgery was performed. Meanwhile, for patients who underwent surgery after neoadjuvant therapy, lymph node positivity was defined by the residual tumor cell, and lymph nodes with evidence of treatment response but no tumor cells were also defined as metastatic nodes in our research.

### Pathological Type

Pathological type was determined based on American Society of Clinical Oncology (ASCO)/College of American Pathologists (CAP) guidelines. Receptor status was considered positive if 10% of cells were stained positive by IHC staining (11); HER2 positive status was defined as 3+. A value of 2+ for HER2 amplification was then confirmed by fluorescence *in situ* hybridization (12). Three subtypes of breast cancer were finally distinguished for analysis based on receptor status (1): HER2+, (2) ER/PR+HER2-, (3) ER/PR-HER2- (13).

### Statistical Analysis

To explore the potential predictive ability, we conducted receiver operating characteristic (ROC) analysis and calculated sensitivity and specificity. An analysis of variance (ANOVA) was used to compare the C-statistic among different imaging models, including mammography, ultrasound, MRI, and ultrasound+MRI models. (Analyzing receiver operating characteristic curves using SAS: Cary, NC: SAS Press 2007.)

## Result

### Demographics

A total of 2,340 patients were enrolled in this research And 1,170 lymph node positive patients were brought into experiment group, while the other 1,170 lymph node negative patients were age- and molecular subtype-matched into control groups. The baseline characteristics showed that age, menopausal age, height, weight, and the molecular subtype in the experiment group and control group were basically balanced (**Table 1**). 53.7% of cases in the experiment group and 53.6% of cases in the control group were Her2+; 33.4% of cases in the experiment group and 33.9% of cases in the control group were ER/PR+, Her2-; only 12.9% of cases in the experiment group and 12.5% of cases in the control group were ER/PR-, Her2-; 21.3% of patients in experiment group and only 4% of cases in control group received neoadjuvant chemotherapy. In total, the true positive rate of ultrasound in detecting lymph node properties reached 62.9% and the false positive rate was 26.6%. The true positive rate of mammography was only 22.2% and the false positive rate was 11.7%. The true positive rate of MRI reached 67.9% while the false positive rate was 33.1%.

**Table 1:** Baseline characteristics of study participants.

	Case (n = 1170)	Control (n = 1170)
Age^*^	51.3 (11.1)	51.4 (11.1)
Menopausal Age	46.7 (6.7)	47.3 (6.7)
Height	160.3 (4.6)	159.8 (4.6)
Weight	61.3 (9.0)	59.9 (8.8)
Menopause, %	50.5	51.6
Pathologic type		
Her2+%	53.7	53.6
ER/PR+Her2- %	33.4	33.9
ER/PR-Her2-%	12.9	12.5
Neoadjuvant chemotherapy (positive)%	21.3	4.0
Ultrasound (positive) %	62.9	26.6
Mammogram (positive) %	22.2	11.7
MRI (positive) %	67.9	33.1

Values are means (SD) for continuous variables; percentages for categorical variables, and are standardized to the age distribution of the study population.

*Value is not age adjusted.

### Differences in Axillary Lymph Node Identification in Total Population by Different Imaging Examinations

**Figure 1:**
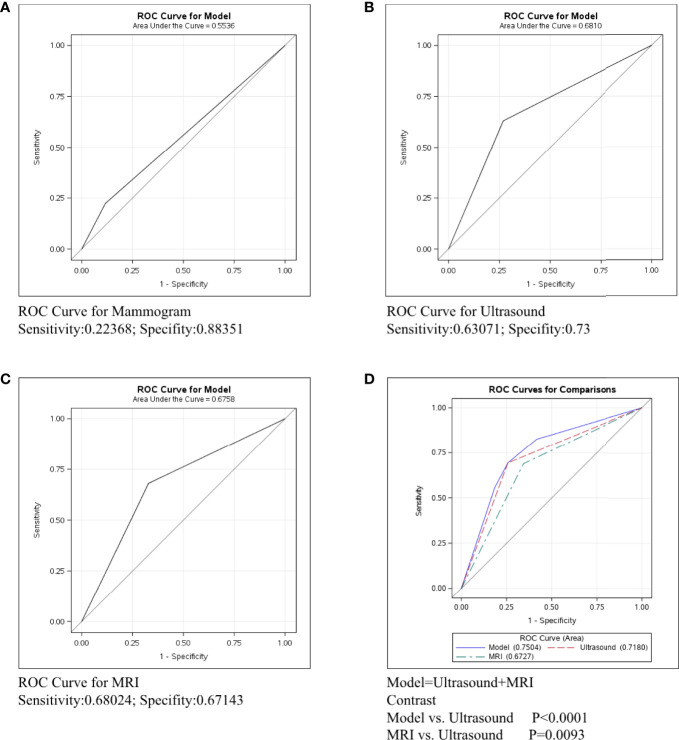
ROC curve analysis for specific imaging examination in all molecular subtypes. The vertical axis is sensitivity, the horizontal axis is 1-specificity. AUC is a parameter used to measure the value of imaging examination in the prediction of axillary lymph nodes. **(A)** ROC curve for mammogram. **(B)** ROC curve for ultrasound. **(C)** ROC curve for MRI. **(D)** ROC curve for ultrasound+MRI.

To assess the predictive ability for axillary lymph node of mammography, ultrasound, and MRI, we calculated the sensitivity, specificity, and C-statistic using receiver operating characteristic (ROC) analysis. Mammography is a common imaging exam used for breast cancer screening and nearly every breast cancer patient would have one before surgery. The sensitivity of mammography was only 0.22368 while the specificity was 0.88351 and the C-statistic was 0.5536 (**Figure 1A**). Ultrasound is another common imaging examination in breast disease. The sensitivity and specificity of ultrasound were 0.63071 and 0.73 respectively, and the C-statistic was 0.6810(**Figure 1B**). The third imaging exam was MRI but it is not as commonly applied for breast cancer patients. The ROC Curve for MRI is explicated in **Figure 1C**, the sensitivity shows 0.68024, the specificity shows 0.67143 and the C-statistic show 0.6758. In **Figure 1D**, we compared the C-statistic of ultrasound, MRI, and ultrasound+MRI. It was found that ultrasound + MRI had the largest C -statistics, while MRI alone had the smallest. The C-statistic was statistically different for MRI and ultrasound (p=0.0093), as well as for ultrasound+MRI and ultrasound alone (p<0.0001).

### Differences in Axillary Lymph Nodes Identification in Her-2+ Patients by Different Imaging Examinations

**Figure 2:**
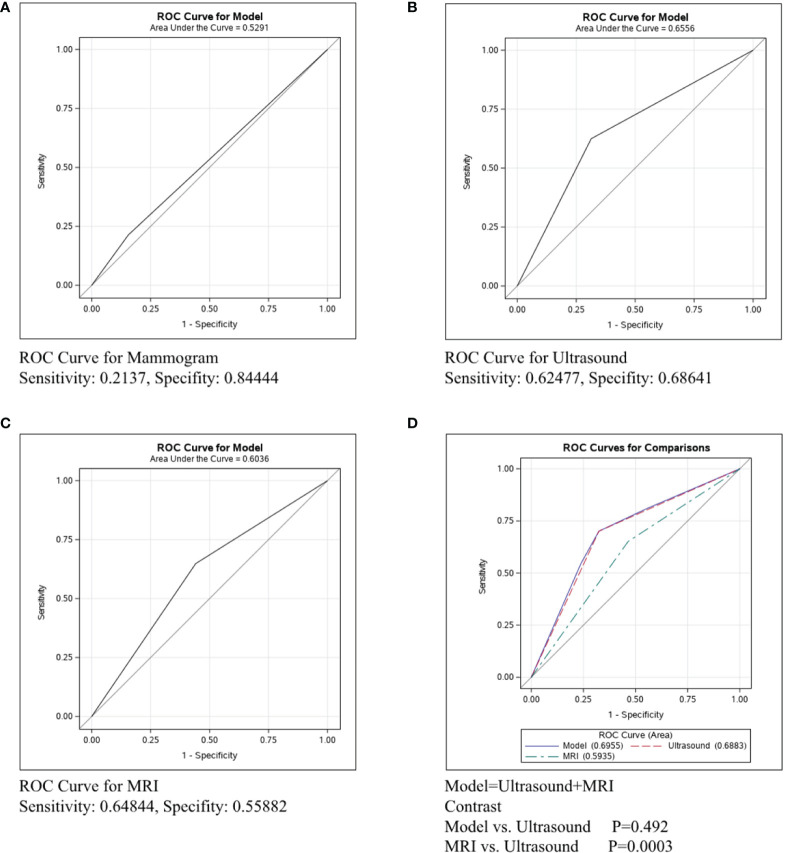
ROC curve analysis for specific imaging examination in Her2+ subtype. The vertical axis is sensitivity, the horizontal axis is 1-specificity. AUC is a parameter used to measure the value of imaging examination in the prediction of axillary lymph nodes. **(A)** ROC curve for mammogram. **(B)** ROC curve for ultrasound. **(C)** ROC curve for MRI. **(D)** ROC curve for ultrasound+MRI.

Breast cancer was divided into three types: Her2+, ER/PR+Her2-, and ER/PR-Her2-. We next conducted ROC curve in the Her2+ subtype to compare the predictive ability of mammography, ultrasound, and MRI. **Figure 2A** shows that the sensitivity of mammography was 0.2137, the specificity was 0.84444, and the C-statistic was 0.5291. **Figure 2B** shows that the sensitivity of ultrasound was 0.62477, the specificity was 0.68641, and the C-statistic was 0.6556. The ROC Curve for MRI is shown in **Figure 2C**, the sensitivity was 0.64844, the specificity was 0.55882, and the C-statistic was 0.6036. In **Figure 2D**, we compared the C-statistics of ultrasound, MRI, and ultrasound+MRI. There was a statistical difference between MRI and ultrasound (P=0.0003). However, no statistical difference was found between ultrasound+MRI and ultrasound alone(p=0.492).

### Differences in Axillary Lymph Nodes Identification in ER/PR+Her2-Patients by Different Imaging Examinations

**Figure 3:**
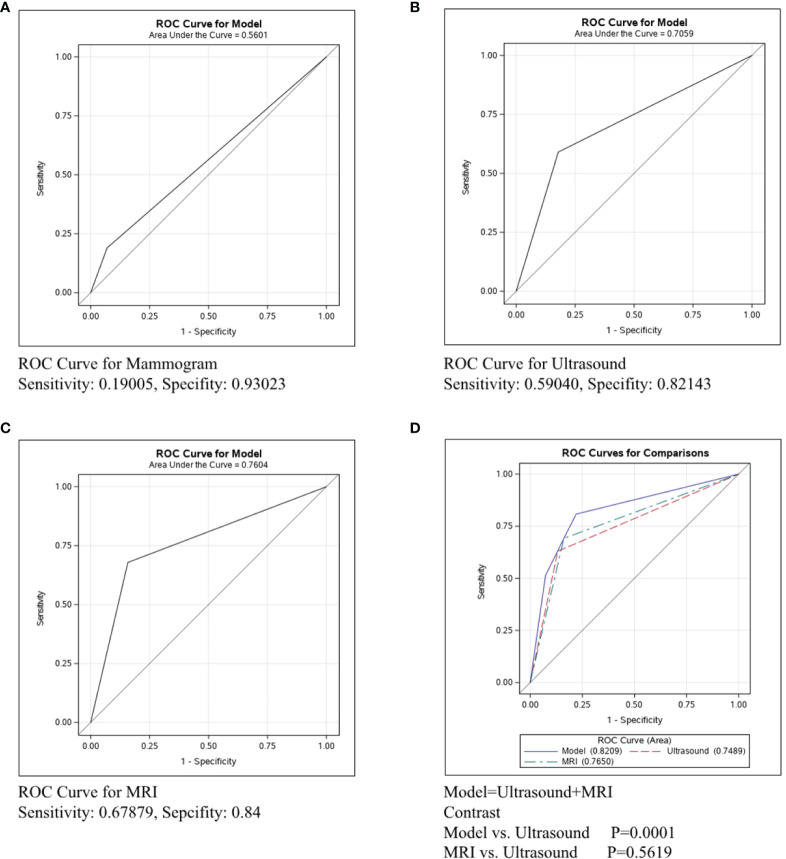
ROC curve analysis for specific imaging examination in ER/PR+Her2-subtype. The vertical axis is sensitivity, the horizontal axis is 1-specificity. AUC is a parameter used to measure the value of imaging examination in the prediction of axillary lymph nodes. **(A)** ROC curve for mammogram. **(B)** ROC curve for ultrasound. **(C)** ROC curve for MRI. **(D)** ROC curve for ultrasound+MRI.

The ROC curve was conducted in the ER/PR+Her2- subtype to compare the predictive ability of mammography, ultrasound, and MRI in **Figure 3**. **Figure 3A** shows that the sensitivity of mammography was 0.19005, the specificity was 0.93023, and the C-statistic was 0.5601. **Figure 3B** shows that the sensitivity of ultrasound was 0.59040, the specificity was 0.82143, and the C-statistic was 0.7059. The ROC Curve for MRI is shown in **Figure 3C**, and the sensitivity was 0.67879, the specificity was 0.84, and the C-statistic was 0.7604. In **Figure 3D**, we compared the C-Statistics of ultrasound, MRI, and ultrasound+MRI. Although no statistical difference was found between MRI and ultrasound (p=0.5619), there was a statistical difference between ultrasound+MRI and ultrasound alone (p=0.0001).

### Differences in Axillary Lymph Nodes Identification in ER/PR-Her2-Patients by Different Imaging Examinations

**Figure 4:**
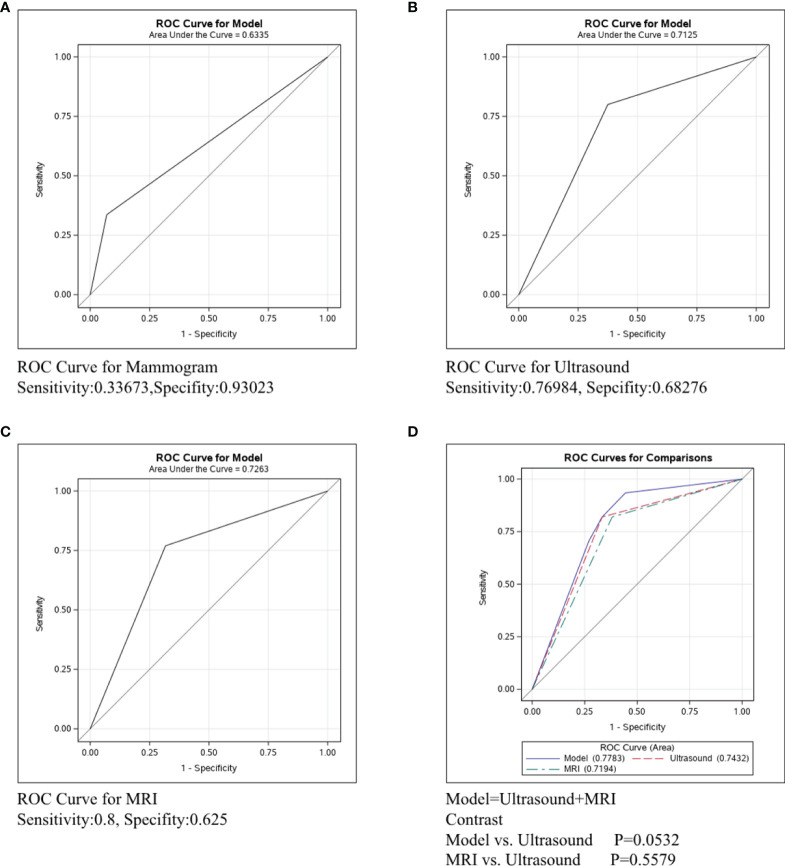
ROC curve analysis for specific imaging examination in ER/PR-Her2- subtype. The vertical axis is sensitivity, the horizontal axis is 1-specificity. AUC is a parameter used to measure the value of imaging examination in the prediction of axillary lymph nodes. **(A)** ROC curve for mammogram. **(B)** ROC curve for ultrasound. **(C)** ROC curve for MRI. **(D)** ROC curve for ultrasound+MRI.

In **Figure 4**, the ROC curve was conducted in the ER/PR-Her2- subtype to compare the predictive ability of mammography, ultrasound, and MRI. **Figure 4A** shows that the sensitivity and specificity of mammography were 0.33673 and 0.93023, respectively, and the C-statistic was 0.6335. **Figure 4B** shows the ROC curve of the ultrasound and the sensitivity and specificity were 0.76984 and 0.68276, respectively, and the C-statistic was 0.7125. **Figure 4C** shows the sensitivity of MRI was 0.8, the specificity was 0.625, and the C-statistic was 0.7263. In **Figure 4D**, we also compared the C-Statistics of ultrasound, MRI, and ultrasound+MRI. However, there was no statistical difference between MRI and ultrasound alone (p=0.5579) and also no statistical difference between ultrasound+MRI and ultrasound alone(p=0.0532).

### Differences in Axillary Lymph Node Identification in Different Molecular Types by Specific Imaging Examination

**Figure 5:**
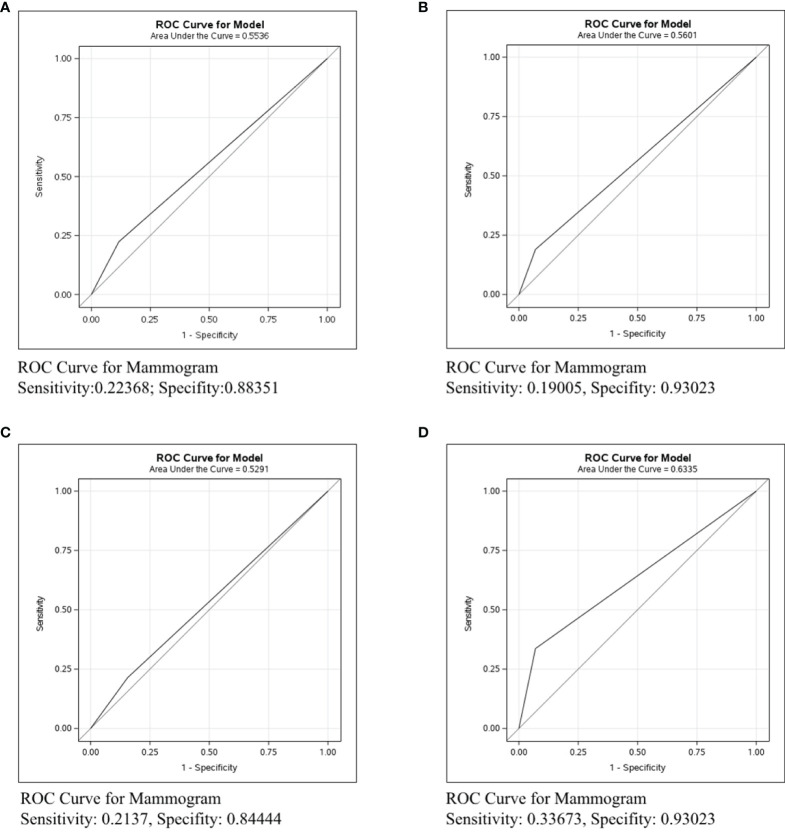
ROC curve analysis for Mammogram in all molecular subtypes. The vertical axis is sensitivity, the horizontal axis is 1-specificity. AUC is a parameter used to measure the value of mammogram in the prediction of axillary lymph nodes. **(A)** ROC curve in all molecular subtypes. **(B)** ROC curve in ER/PR+Her2-negative subtype. **(C)** ROC curve in Her2+ subtype. **(D)** ROC curve in ER/PR-Her2- subtype.

**Figure 6:**
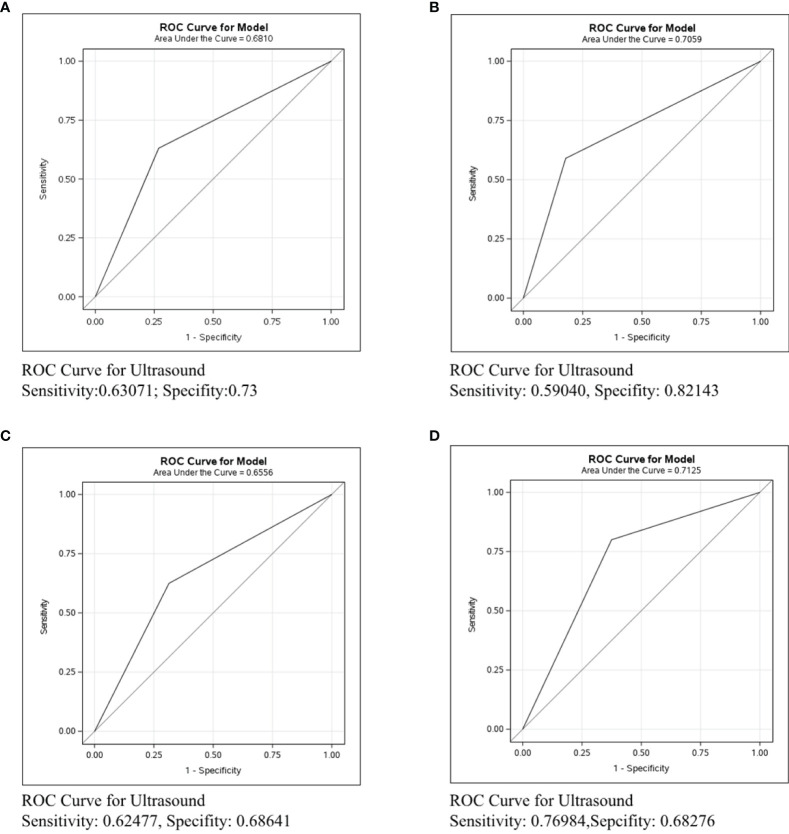
ROC curve analysis for Ultrasound in all molecular subtypes. The vertical axis is sensitivity, the horizontal axis is 1-specificity. AUC is a parameter used to measure the value of ultrasound in the prediction of axillary lymph nodes. **(A)** ROC curve in all molecular subtypes. **(B)** ROC curve in ER/PR+Her2- subtype. **(C)** ROC curve in Her2+ subtype. **(D)** ROC curve in ER/PR-Her2- subtype.

**Figure 7:**
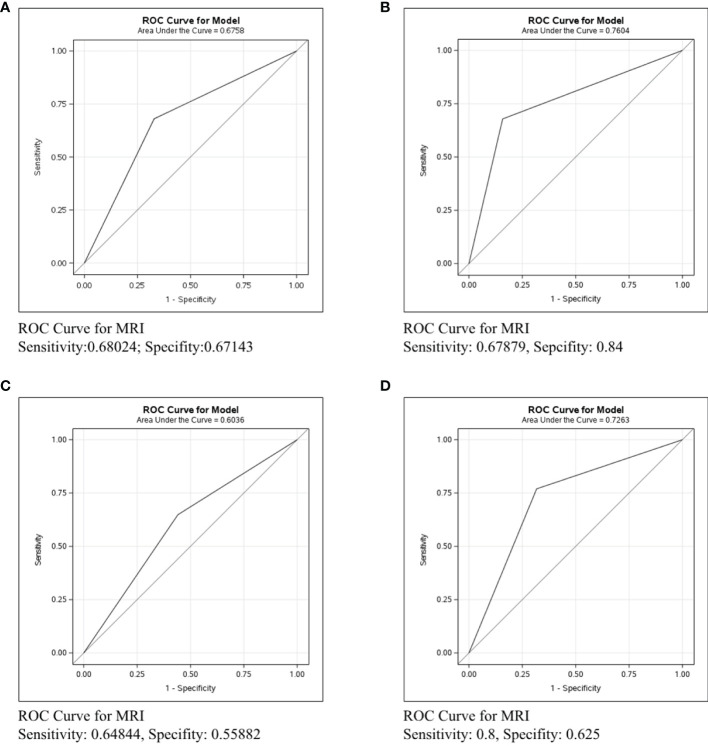
ROC curve analysis for MRI in all molecular subtypes. The vertical axis is sensitivity, the horizontal axis is 1-specificity. AUC is a parameter used to measure the value of MRI in the prediction of axillary lymph nodes. **(A)** ROC curve in all molecular subtypes. **(B)** ROC curve in ER/PR+Her2- subtype. **(C)** ROC curve in Her2+ subtype. **(D)** ROC curve in ER/PR-Her2- subtype.

The accuracy of each imaging examination in different molecular subtypes was also compared. Mammography had the worst predictive power in assessing axillary lymph node status in breast cancer of any molecular type, as shown in **Figure 5**. The C-statistics were 0.5291, 0.5601, and 0.6335, respectively. **Figure 6** shows the accuracy of ultrasound, which was the best in ER/PR-Her2- patients with the C-statistic of 0.7125. In ER/PR+Her2- patients, the accuracy was next to that in ER/PR-Her2- patients, with the C-statistic of 0.7059. In Her2+ patients, accuracy was the worst, with the C-statistic of only 0.6556. **Figure 7** shows MRI had the best accuracy with a C-statistic of 0.7604 in ER/PR+Her2 -patients, while the worst accuracy was in HER2+ patients with the C-statistic of 0.6036. MRI accuracy in ER/PR-Her2- patients was moderate with the C-statistic of 0.7203.

## Discussion

The progression of cancer is characterized by metastasis. As the first organ to be involved during metastasis, the presence of regional lymph node metastasis correlates with dissemination to distant organs and a poorer prognosis (1, 14). Based on preoperative imaging evaluation of axillary, clinicians would choose fine needle aspiration, core needle biopsy, SLNB, or ALND to treat potential local metastases. Historically, ALND was regarded as the most accurate and radical method for assessing and controlling regional metastatic spread (2). However, excessive treatment would bring unnecessary pain for pathologically node-negative patients, while residual lesions would bring potential recurrent risk. Therefore, accurate preoperative imaging evaluation of axillary lymph node involvement is very important for precision treatment of breast cancer patients.

The imaging methods we reviewed to assess the metastasis of axillary lymph nodes included mammography, ultrasound, and MRI. Mammography is the standard imaging modality for breast cancer screening, especially for postmenopausal women whose breast are almost entirely fatty (2, 15). However, our research showed that mammography was not reliable for the evaluation of lymph node metastasis (**Figure 1A**), This poor prediction may be from extremely low sensitivity but high specificity, which was consistent with the results from former studies (2). The low sensitivity may be attributed to the limited spatial resolution and the fact that the axillary area may not be fully visualized. Nevertheless, the high specificity of mammography can help raise the suspicion of malignancy detected by ultrasound or MRI. Ultrasound is usually the preferred method for the assessment of lymph node involvement in breast cancer patients (2, 16). It was reported that the sensitivity of ultrasound had a wide range, between 49% and 87%, and the specificity was between 55% and 97% (2). Our study reached a similar conclusion (**Figure 1B**). In the identification of lymph node metastasis, the evaluation standards include the size criteria as well as the morphologic criteria. Moreover, the Color Doppler allows for the visualization of intranodal vascular pattern and the abnormal cortical blood flow to help further increase the detection rates (17). MRI has a minor role in the diagnosis of breast cancer and metastatic lymph node in a clinical setting, mostly because of its high price and time-consuming features (18, 19). According to the literature, the pooled diagnostic sensitivity and specificity of MRI to detect axillary lymph node metastasis in patients with breast cancer were 75%-80% and 89%-91% respectively (19). As our research suggests, the sensitivity and specificity of MRI in detecting metastatic nodes were both weaker than ultrasound (**Figures 1B–D**), mainly because the dedicated breast coils may limit the complete visualization of the axilla. Moreover, the pulsation artifact from heart may occasionally obscure the images of lymph nodes (20).

Molecular subtype classification of breast cancer is a regular process for individualized cancer management. Previous studies have indicated that the molecular subtype was correlated with characteristic imaging manifestation of the lump (21). Therefore, we next explored whether the imaging diagnostic performance of lymph nodes differ among different molecular subtypes of breast cancer.

**In Her2+ subtype,** the C-statistics of mammography, ultrasound, and MRI were 0.5291, 0.6556 and 0.6036, respectively (**Figures 2A–C**). Clearly, ultrasound was the most precise examination for lymph node assessment. Moreover, no statistical difference was found between ultrasound+MRI and ultrasound alone for detecting metastatic lymph nodes (p=0.492, **Figure 2D**). To sum up, ultrasound was the most recommended examination in Her2+ patients and MRI was not strictly necessary for the diagnosis lymph node involvement in HER2+ breast cancer.

**In ER/PR+Her2- patients**, the C-statistics of mammography, ultrasound, and MRI were 0.5601, 0.7059, and 0.7604, respectively (**Figures 3A–C**). Our study indicates that the diagnostic effect of MRI and ultrasound were similar (p=0.5619, **Figure 3D**), while ultrasound+MRI increased the accuracy for lymph node assessment than ultrasound alone (p=0.0001, **Figure 3D**). We recommend ultrasound+MRI in ER/PR+Her2- patients for more accurate axillary assessment. Currently, since there are harmful side-effects of axillary surgery, minimizing, and even eliminating the axillary surgery is a clear trend. Related clinical trials include BOOG 2013-08 trial (22), SOUND trial (23),and INSEMA trial (24). According to the literature, less than 4 involved nodes (1-3 macro-metastases) and were considered to have little influence in breast cancer mortality, in which condition and the risk of disease progression depended mainly on the biological characteristic of the primary tumor (24). Based on this, to positively decrease the axillary side effect rates and improve the quality of life, the axillary surgery should be considered mainly on the basis of tumor traits rather than node involvement. As we know, the prognosis of ER/PR+Her2- subtype is best among three subtypes (25). Meanwhile, the diagnostic accuracy of ultrasound+MRI in lymph node metastases was also highest in the ER/PR+Her2- subtype in our study. Therefore, we can reasonably assume that the lymph node negative ER/PR+Her2- patients diagnosed by imaging tests would rarely have massive positive lymph node pathologically(≥4 macro-metastases), and compared with axillary surgery, no axillary surgical intervention for clinically node negative breast cancer would bring non-inferior overall survival rates and better quality of life. In the future, we would like to design prospective studies with ER/PR+Her2- patients to explore the subtraction of axillary surgery in patients with negative lymph nodes by adequate imaging evaluation.

**In ER/PR-Her2- patients**, the C-statistics of mammography, ultrasound and MRI were 0.6335, 0.7125 and 0.7263, respectively (**Figures 4A–C**). The diagnosis effect of MRI and ultrasound was similar (p=0.5579, **Figure 4D**), while adding MRI did not increase the accuracy for lymph node assessment by ultrasound (p=0.0532, **Figure 4D**). Nonetheless, we can see a trend that adding MRI improved accuracy, and perhaps increasing the sample size could get a statistical difference (26). Therefore, in ER/PR-Her2- patients, ultrasound was the preferred imaging examination and if cost is not a regard, MRI examination may be also feasible. Next, we performed horizontal comparison. The lymph node assessment accuracy of mammography, ultrasound, and MRI were all worse in the Her2+ subtype than in ER/PR+Her2- or ER/PR-Her2- subtypes. In order to improve the detection rate of metastasis lesion, new technologies for axillary assessment such as contrast-enhanced ultrasonography (27), digital breast tomosynthesis (DBT) (28), and the lymph PET (29) should be further explored, with an expected increase in the accuracy and predictability of axillary lymph nodes and increase in the benefit to more patients.

For the first time, our study explored the influence of breast cancer molecular subtypes on the diagnostic performance of three different axillary imaging. However, our research was a single center and retrospective study. The amount of data in hierarchical analysis is relatively small and a prospective study with a larger sample size is expected in the future.

## Conclusion

From a clinical perspective, our job reviewed the diagnostic performance of three commonly used axillary imaging methods in different molecular subtypes of breast cancer. It may give some suggestion in the selection of lymph node assessment examinations and the subsequent axillary treatments. ER/PR+Her2- breast cancer may become a breakthrough in research on reducing axillary lymph node surgery due to its high imaging accuracy and good prognosis.

## Data Availability Statement

The raw data supporting the conclusions of this article will be made available by the authors, without undue reservation.

## Ethics Statement

Research involving human subjects complied with all relevant national regulations, institutional policies and is in accordance with the tenets of the Helsinki Declaration, and has been approved by the Medical Ethics Review Committee of the first affiliated hospital of Nanjing Medical University (reference number 2021-SR-182, Figure S5.

## Author Contributions

YX, YC and XH collected data. YH and JP analyzed the data. MZ, XH and SW designed the experiments. MZ and YH writed the paper and approved the submission and publication.

## Funding

This study was financially supported by the National Natural Science Foundation of China (81702607, 81672612), Scientific Research Project of Jiangsu Province Association of Maternal and Child Health (FYX202018), Scientific Research Project of Jiangsu Women and Children Health Hospital for Youth Talents (FYRC202003).

## Conflict of Interest

The authors declare that the research was conducted in the absence of any commercial or financial relationships that could be construed as a potential conflict of interest.

## Publisher’s Note

All claims expressed in this article are solely those of the authors and do not necessarily represent those of their affiliated organizations, or those of the publisher, the editors and the reviewers. Any product that may be evaluated in this article, or claim that may be made by its manufacturer, is not guaranteed or endorsed by the publisher.

## Supplementary Material

Supplementary Figure 1 Flowchart of the patient selection process in this study.

Supplementary Figure 2 Morphological features of mammograph that are predictors of lymph node malignancy. Size>2cm **(A, C)**, increased density **(B)**, rounded or irregular shape **(B, D)**, spiculated margins or the absent fatty hilum).

Supplementary Figure 3 Ultrasonic morphological features that can predict lymph node malignancy. Focally or diffusely thickened cortex (> 3 mm thick) **(A, B)**, deformed or absent fatty hilum **(C, D)**, abnormal blood flow **(B, D)**.

Supplementary Figure 4 Morphological features of MRI that can predict lymph node malignancy. Irregular contour compared with the contralateral axilla **(A–C)**, node measuring greater than 1 cm **(A, B**, thickened cortex >3 mm **(A, B)**, the loss of fatty hilum **(C)** Supplementary Figure 5 Ethics review.
