# Supplementary figures and images for: Optimal Selection of Imaging Examination for Lymph Node Detection of Breast Cancer With Different Molecular Subtypes
Source: Front Oncol. 2022 Jul 13;12:762906. doi: 10.3389/fonc.2022.762906 (PMC9326026; doi:10.3389/fonc.2022.762906)

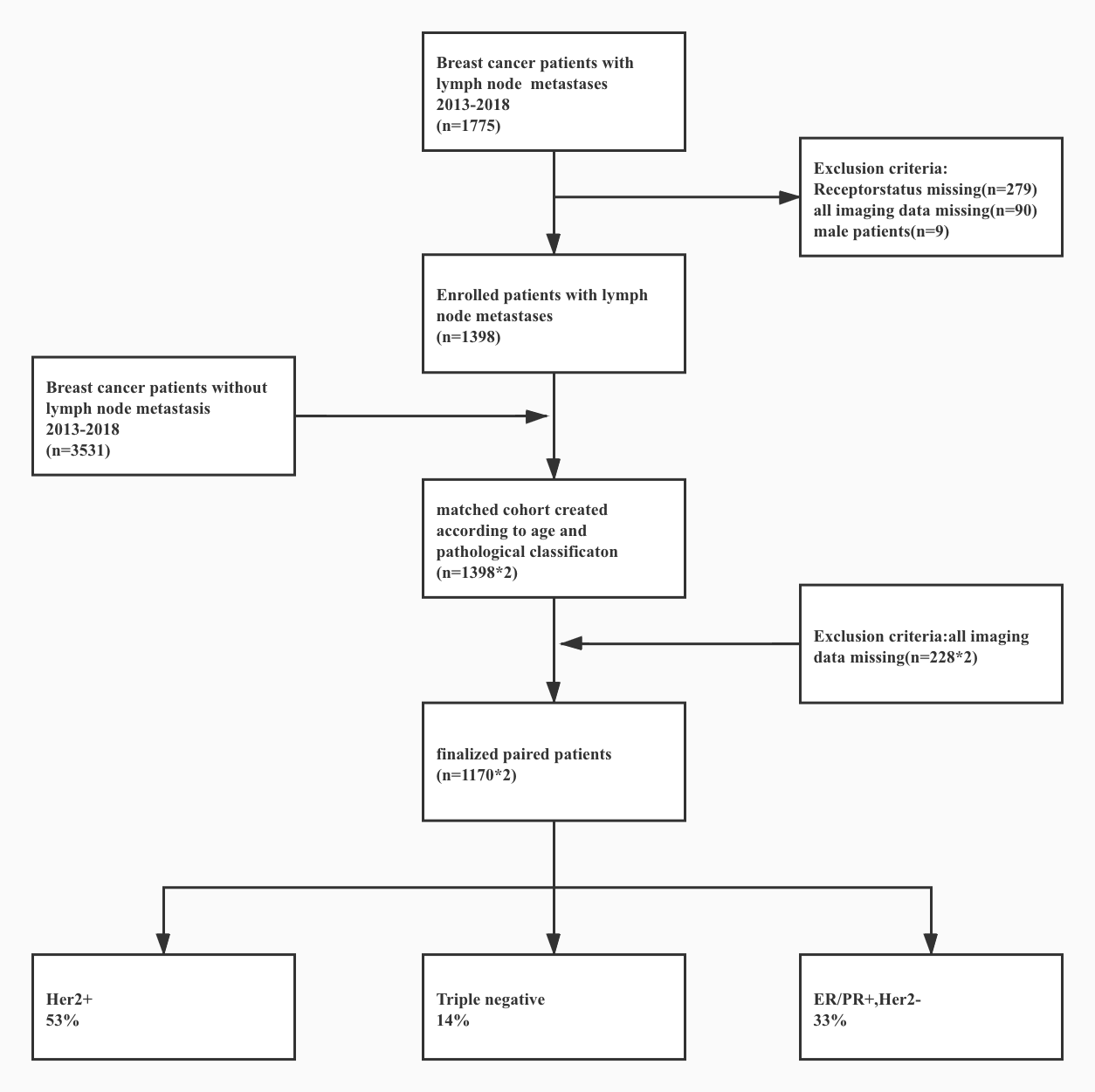

Supplement: Supplementary Figure 1 — Flowchart of the patient selection process in this study. [file Image_1.jpeg]

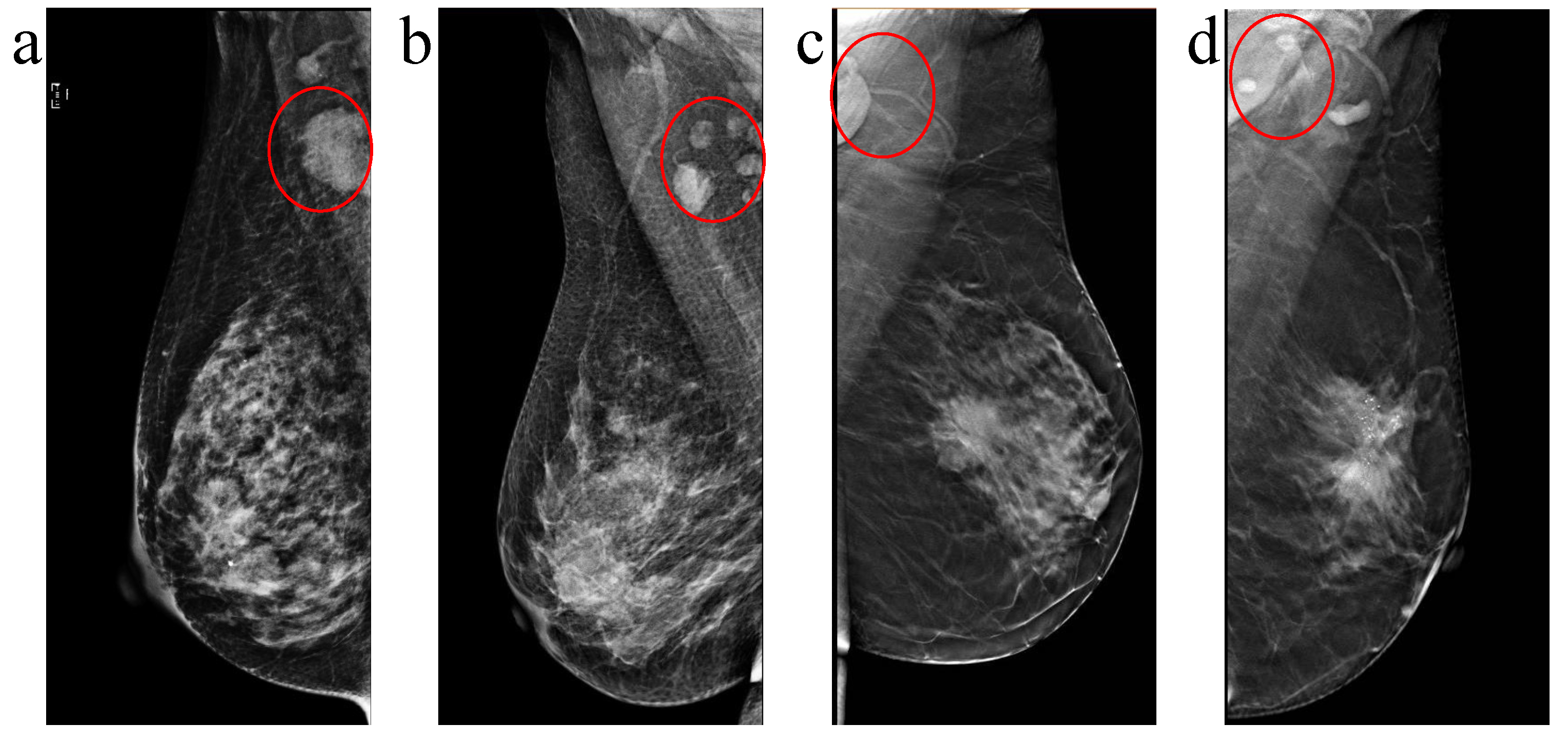

Supplement: Supplementary Figure 2 — Morphological features of mammograph that are predictors of lymph node malignancy. Size>2cm (A, C) , increased density (B) , rounded or irregular shape (B, D) , spiculated margins or the absent fatty hilum). [file Image_2.jpeg]

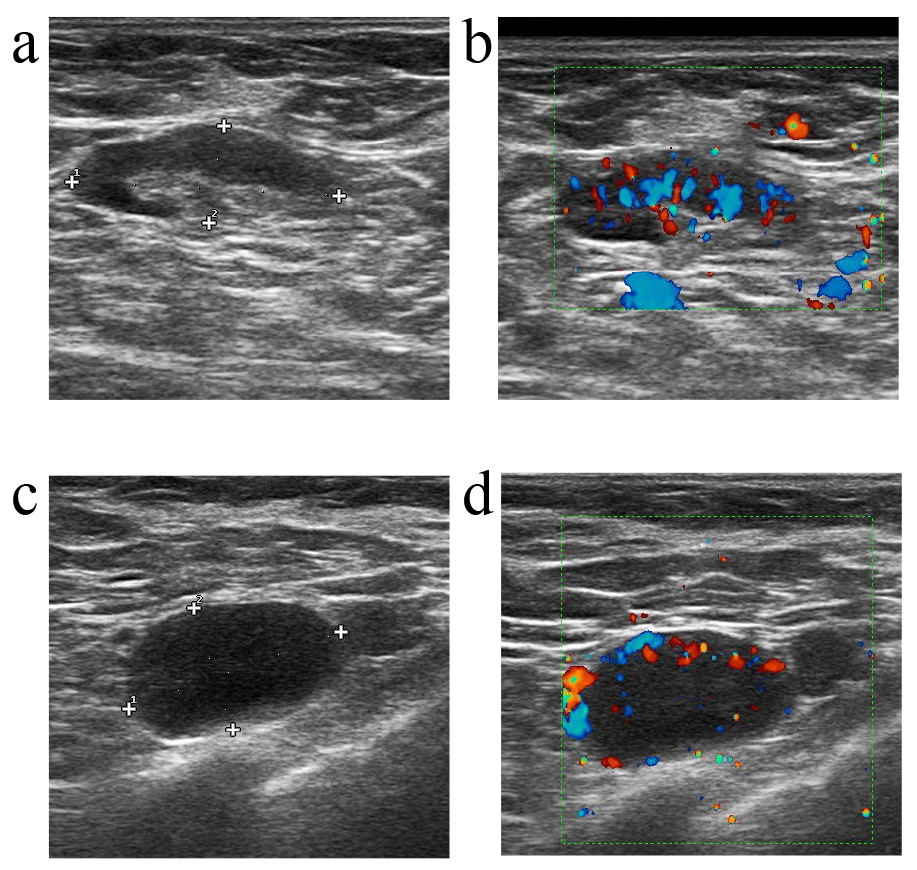

Supplement: Supplementary Figure 3 — Ultrasonic morphological features that can predict lymph node malignancy. Focally or diffusely thickened cortex (> 3 mm thick) (A, B) , deformed or absent fatty hilum (C, D) , abnormal blood flow (B, D) . [file Image_3.jpeg]

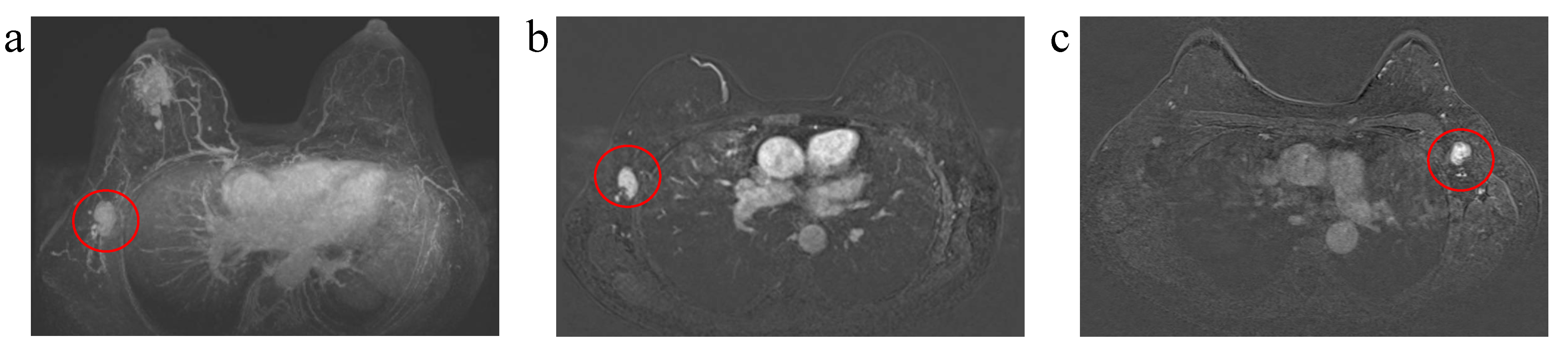

Supplement: Supplementary Figure 4 — Morphological features of MRI that can predict lymph node malignancy. Irregular contour compared with the contralateral axilla (A–C) , node measuring greater than 1 cm (A, B , thickened cortex >3 mm (A, B) , the loss of fatty hilum (C) Supplementary Figure 5 Ethics review. [file Image_4.jpeg]
